# Community-based deworming for hookworm benefits children treated within schools and is cost-effective due to economies of scale: the CoDe-STH trial

**DOI:** 10.1016/j.lanwpc.2023.100949

**Published:** 2023-10-31

**Authors:** Goylette F. Chami

**Affiliations:** Big Data Institute, Nuffield Department of Population Health, University of Oxford, Oxford, UK

The treatment of infections caused by soil-transmitted helminths (STHs), including roundworm, whipworm, and hookworm, are managed through mass drug administration (MDA). These large-scale blanket treatment programmes provide typically donated medicines without diagnosis and at no cost to at-risk individuals in areas with known STH prevalence. The distributional strategy and frequency of treatment is determined by prevalence cutoffs set by the World Health Organization (WHO). School-aged children (e.g. 5–15 years) are the target population for treatment due to the believed burden of infection being concentrated in this age group.^1^ In efforts to reach this target population most easily, medicines are distributed through primary schools. However, treatment through communities remains important. Children may not be enrolled in school and younger children are at-risk of infection. For hookworm, adults have been shown to harbour the heaviest burden of infection and may act as reservoirs of infection to sustain transmission in communities.^2^ Criteria for determining whether school or community-based MDA should be conducted for STHs ultimately depend on the anticipated reduction in infection prevalence/intensity and cost-effectiveness of each distributional strategy.

Recently published in *The Lancet Regional Health—Western Pacific,* the CoDe-STH trial in Vietnam^3^ contributes to the growing body of evidence supporting community-based MDA for hookworm. CoDe-STH extends established results from meta-analyses^4^ and other large-scale trials such as TUMIKIA (Kenya),^5^ Deworm3 (India, Malawi, and Benin),^6^ and Geshiyaro (Ethiopia).^7^ Sixty-four schools were enrolled in CoDe-STH. Children aged 6–11 years attending all study schools in grades 1–4 were eligible to receive a single dose of 400 mg of albendazole. Schools were randomized 1:1 where the control arm treated children within schools only and the intervention arm treated not only children within schools but also treated all members aged 1+ years from hamlets/communities served by schools in the interventional arm. *Necator americanus* prevalence and intensity were measured from qPCR on one stool sample as the primary and secondary outcomes, respectively, 12 months after treatment. The trial was underpowered to find differences in hookworm prevalence, although shown to be impacted by community-based MDA elsewhere in the TUMIKIA trial in Kenya.^5^ However, infection intensity, as converted to eggs per gram of stool from qPCR cycle thresholds, differed between study arms. Children treated within schools where their home communities also were treated had a 56.0% reduction in mean *N. americanus* infection intensity compared to only a 3.4% reduction found in children who were treated in schools where their home communities were not offered treatment. Baseline infection intensity was similar between study arms. The key implications of these results are that children benefit, even when treated within schools, from pre-school aged children, non-enrolled school children, and adults being treated within their communities. Additional studies are needed to understand whether this finding directly supports the role of untreated individuals in sustaining community transmission, and in turn, predisposing WHO target populations to reinfection.

In the same issue of *The Lancet Regional Health—Western Pacific*, Trinos and colleagues^8^ present a detailed cost survey of the CoDe-STH trial and use these costs as inputs to compare the general cost-effectiveness of MDA within the community (full population) versus within only schools (enrolled children aged 5–14 years) for STHs in Dak Lak, Vietnam. This model considers the donation of medicines for school-based MDA and the direct purchase of medicines for community-based MDA. Community-based MDA versus school-based MDA was estimated to cost $472,000 and $117,000 per year, respectively. The target populations for which the costs were estimated included 283,123 children, and 1,886,230 community members. The economic cost per person for a government payer was $0.27 for community-based MDA compared to $0.43 for school-based MDA. Over 10 years in a static model, the additional estimated disability-adjusted life years (DALYs) averted with community-based MDA was 121,465 with a cost of $28.55/DALY. Cost-effectiveness of community-based MDA was due to economies of scale—as treatment programmes expand and treat more people there are administrative costs that are not reincurred or that max out and the incremental cost of treating another person decreases as more people are treated. Other studies^9^^,^^10^ have shown that cost-effectiveness can be influenced by STH prevalence and consideration of costs for supportive activities such as advocacy campaigns.

The CoDe-STH trial provides important insights into the considerations needed and challenges for large-scale, community-based evaluation of hookworm deworming campaigns. However, these findings leave open several questions. Future work still is needed to generalise to other STHs and to identify prevalence thresholds for reducing transmission. Ongoing comparisons of distributional strategies are underway to investigate the feasibility of reducing STH prevalence below 2%^6^ and the relative impact of MDA against or coupled with alternative interventions to improve water, sanitation, and hygiene.^7^ To support in-country commitments by resource-constrained governments, future studies might consider comparing the cost-effectiveness of treatment for only one target age group of children through either schools or communities.

## Contributors

GFC conceptualised the commentary, wrote the original draft, and reviewed and edited the commentary.

## Declaration of interests

The authors declare no conflicts of interest.
